# Three-dimensional CT reconstruction for visualization of esophageal varices after injection with combined cyanoacrylate and Lipiodol (with video)

**DOI:** 10.1016/j.igie.2023.04.005

**Published:** 2023-05-11

**Authors:** Calvin Jianyi Koh, Sabrina Xin Zi Quek, Koo Chieh Sian

**Affiliations:** 1 Division of Gastroenterology and Hepatology, National University Hospital, Singapore; 2 Department of Medicine, Yong Loo Lin School of Medicine, National University of Singapore, Singapore; 3 The Gastroenterology Group, Gleneagles Hospital, Singapore

Three-dimensional imaging has been used mainly in orthopedic, spine, and neurosurgical fields, although its use in real-time biliary tree imaging in endoscopy has been reported.^1^ We have similarly found CT reconstructions to be helpful in the assessment of the extent of esophageal varices.

A 41-year-old man with a history of chronic ethanol use presented to the emergency department with massive hematemesis. Initial laboratory results showed a hemoglobin level of 3.8 g/dL, hematocrit of 12.2%, platelet count of 25 × 10^9^/L, and a prothrombin time of 21.5 seconds (normal range, 9.7-11.8). An intravenous somatostatin infusion was initiated; packed red blood cells, platelets, and fresh frozen plasma were transfused; and he was intubated for airway protection.

On urgent upper endoscopy, massive bleeding esophageal varices were seen (Fig. 1). Band ligation was unsuccessful because of scarring (Fig. 2), and injection therapy was performed for hemostasis (Fig. 3) with a combination of .5 mL *N*-butyl-2-cyanoacrylate with .8 mL of Lipiodol (GUERBET, Paris, France) as described by Seewald et al.^2^ This was performed by sequential injection in 1.3-mL aliquots, with reassessment after each injection to reduce the overall volume required. Although the routine use of glue in bleeding esophageal varices is not advocated because of the increased risk of adverse events, in particular glue embolization, this was performed as a salvage procedure because of the severity of the bleeding and the limited options given that the patient was at high risk of hepatic decompensation with a portal pressure–lowering shunt placement.

**Figure 1:**
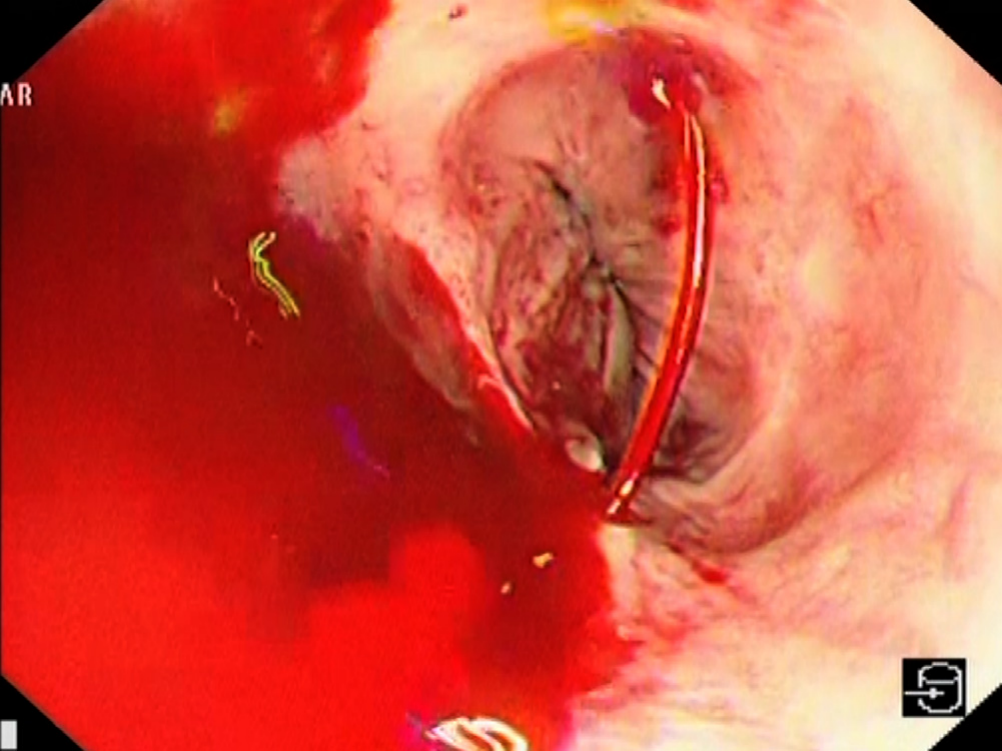
Spurting esophageal varices.

**Figure 2:**
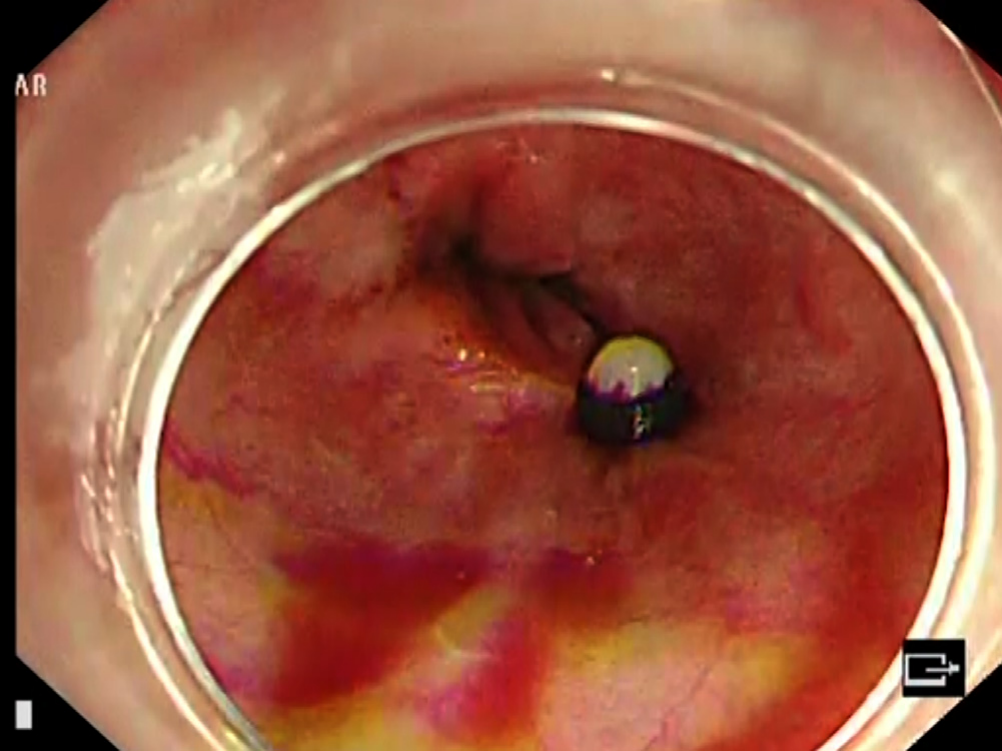
Unsuccessful band placement because of scarring.

**Figure 3:**
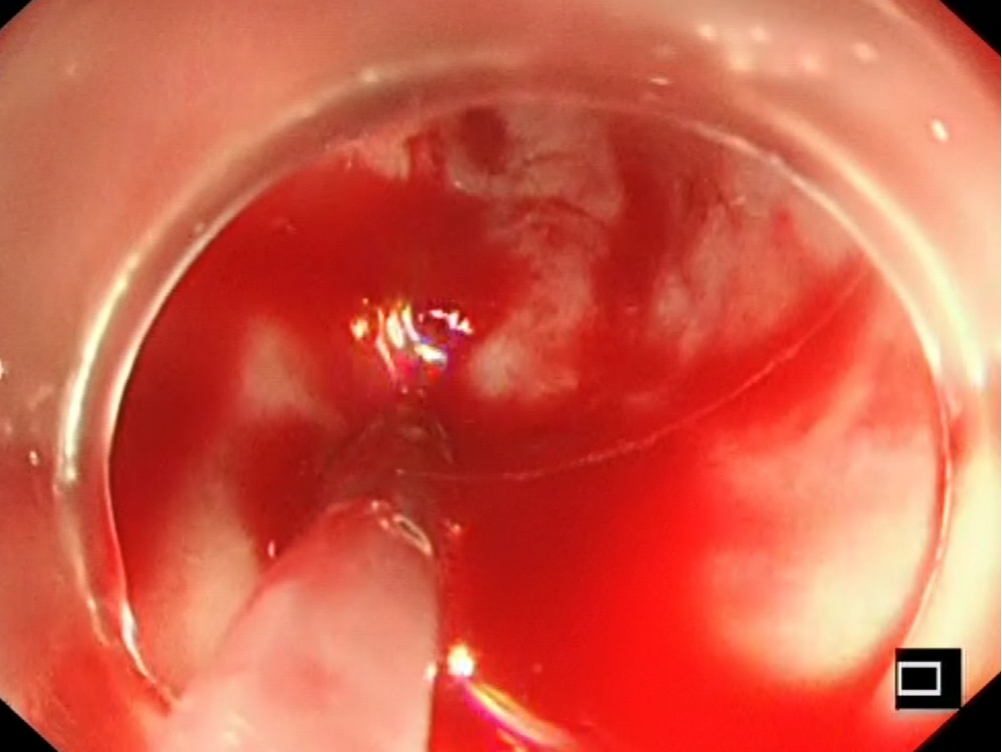
Glue injection.

A subsequent CT performed the next day to evaluate for structural causes of worsening portal hypertension (eg, hepatocellular carcinoma) did not show any such etiology but did demonstrate the distal esophageal portal circulation, which had been successfully occluded with iodine-containing Lipiodol. Video 1 (available online at www.igiejournal.org) and Figure 4 show the 3-dimensional reconstruction of the CT. There was no evidence of distal glue embolization on the CT. The patient recovered and remained well for more than 6 months.

**Figure 4:**
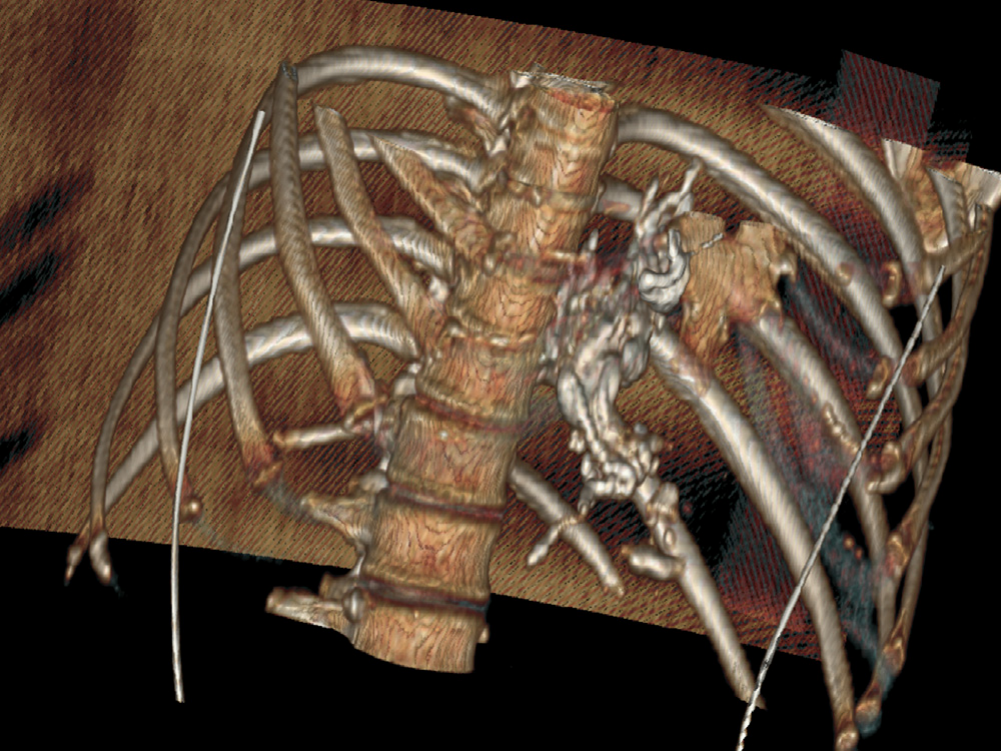
Three-dimensional reconstructed view of the glue outlining the esophageal varices.

The method of *N*-butyl-2-cyanoacrylate and Lipiodol injection is more commonly used for gastric variceal injections,^3^^,^^4^ but this case demonstrates its utility in an emergent case, where as subsequent imaging shows not only the bleeding spot but much of the esophageal portal system was successfully occluded. Esophageal variceal bleeding is a serious adverse event of portal hypertension, and this case is an apt reminder that in bleeding varices, the bleeding spot is often just the tip of the iceberg, and a holistic approach to lowering the portal pressure should be pursued.

## Disclosure

*All authors disclosed no financial relationships.*

## Supplementary data

Video 1 Video showing the endoscopic intervention and the 3D reconstruction.
